# Verification of the Influence of Processing History through Comparing High-Speed Melt Spinning Behavior of Virgin and Recycled Polypropylene

**DOI:** 10.3390/polym14163238

**Published:** 2022-08-09

**Authors:** Wataru Takarada, Mohammad A. Barique, Tatsuma Kunimitsu, Takao Kameda, Takeshi Kikutani

**Affiliations:** 1 Department of Materials Science and Engineering, Tokyo Institute of Technology, Tokyo 152-8550, Japan; 2 Futuristic Technology Department, SANKO GOSEI Ltd., Toyama 939-1852, Japan; 3 School of Materials and Chemical Technology, Tokyo Institute of Technology, Yokohama 226-8503, Japan

**Keywords:** polypropylene, melt spinning, processing history, flow-induced crystallization, recycle, physical deterioration, entanglement, wide-angle X-ray diffraction, a*-axis orientation

## Abstract

A ‘model’ material of recycled polypropylene (PP) was prepared through the injection molding process, and the effect of processing history on the polymer characteristics was investigated through the high-speed melt spinning of virgin and recycled PP. On-line measurement of the thinning behavior of the spin-line revealed the downstream shift of solidification point for the recycled PP at the take-up velocity of 1.0 km/min, indicating the suppression of flow-induced crystallization. The difference was not clear at higher take-up velocities of up to 5 km/min. For any identical take-up velocity, no clear difference in the stress-strain curves and birefringence of the fibers from virgin and recycled PP could be observed, whereas the detailed investigation on the variation of relative amount of *c*-axis and a*-axis oriented crystals in the fibers prepared at varied take-up velocities suggested the deterioration of flow-induced crystallization at 1.0 km/min. It was speculated that the processing history induced the lowering of the entanglement density, which affected the melt spinning and crystallization behavior. An undistinguishable difference between the virgin and recycled PP at increased take-up velocities suggested the existence of an optimum elongational strain rate for the detection of the different states of molecular entanglement.

## 1. Introduction

In recent years, recycling of used polymers has been pointed out as an issue of great importance from the viewpoints of environmental problems [1] and resource protection. Every year over 300 million tons of polymers are being produced worldwide [2], leaving a huge amount of CO_2_ emission [3,4] which is the cause of catastrophic global warming [3,5,6]; however, almost all of the used plastics are discarded to landfills and open-fire burning, and only a very small percentage of them are being recycled in environmentally friendly ways, probably due to the lack of sufficient basic research data for recycling of various types of used plastics.

There are several types of methods for recycling polymer products; however, it depends on the structure and properties of the polymers to be recycled. Among all the recycling methods, mechanical recycling is considered to be the best method in terms of reducing CO_2_ emission, time and cost of processing [7,8,9]. Products of mechanically recycled polymers often are recognized as inferior in performance than their virgin products; however, several recent research results showed that this performance deterioration is not due to their chemical degradation, but rather due to their physical deterioration [10].

Processing history, such as flow, deformation or thermal history, in any polymer material is supposed to have an impact on the structure and properties of a new product from it. Tominaga et al. [7] worked on recycled polypropylene (PP) products and found that the crystal structures and internal structures of recycled polymers are changed due to the molding process. Yamaguchi et al. [11] worked on long-chain branched PP with the processing history of compression molding and found a depression of elasticity. They ascribed it as the result of shear modification through the alignment of long branches to a backbone chain. Khanna et al. found that the morphology and mechanical properties of Nylon 6 molded products depend upon the processing history of its starting Nylon 6 resin [12].

Polymer products are basically composed of several structural elements such as extended chains, folded lamellar crystals, entangled and trapped chains, taut tie molecules, etc. [13]. Mechanical and other related properties of a polymer product are mainly governed by the density of entanglements and taut tie molecules in the polymers [7,14,15]. Tominaga et al. worked on recycled PP and found that physical deterioration of PP was caused by the decrease in tie molecules [16]. They found that the molecular weight of recycled PP was not changed due to recycling, and they were able to recover the physical properties by adopting an appropriate molding condition of the mechanical recycling process.

In recent years, global demand and production of PP have been increasing tremendously [17], due to its applications in various areas. Poly(propylene/ethylene) copolymer with a low content of ethylene (1–10%) is also used widely as structural material [18]. To recycle the used plastics, it is indispensable to understand the effect of processing history in-depth as mentioned above. In the present paper, we conducted the study on the verification of the effect of the processing history of molded and recycled products of poly(propylene/ethylene) copolymer applying the high-speed melt spinning process. Particular attention was paid to the in situ measurement of the processing behavior of virgin and recycled polymers, considering that the difference in the structure and properties of prepared fibers originated from the different processing behavior. The melt spinning process is a steady state process of simple elongational flow with a high elongational strain rate. Through varying the take-up velocity, differences in the viscoelastic flow behavior of molten polymers can be investigated for a wide range of relaxation times. It should be noted that the results of in situ measurement include the information on the structure development behavior, i.e., molecular orientation, and flow-induced crystallization as well.

## 2. Experimental Section

### 2.1. Preparation of Recycled PP Model Materials

**Figure 1:**
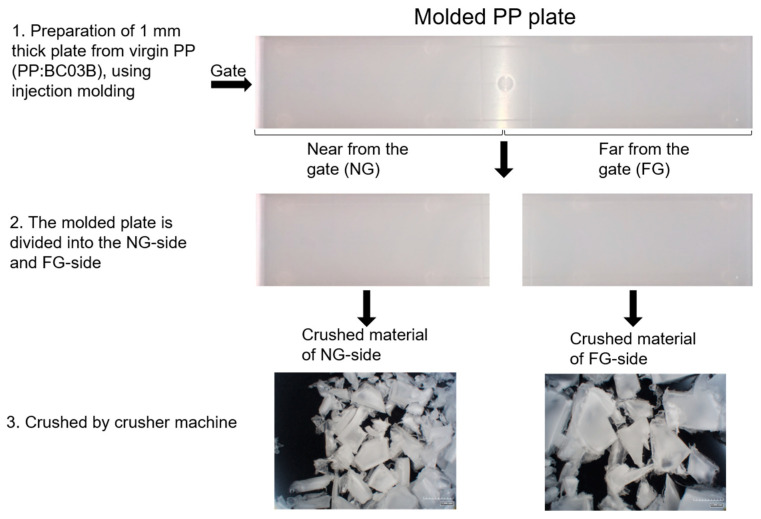
Preparation of recycled PP copolymer for melt spinning.

For the preparation of ‘ideal’ recycled polymer for investigating the effect of processing history on the properties of resultant products, firstly injection molding was performed using the typical polypropylene (PP) pellet in the market, i.e., propylene–ethylene copolymer with propylene composition of 91 wt% (NOVATEC PP (BC03B), MFR = 30 g/10 min, Japan Polypropylene). A flat plate with the length, width and thickness of 200 × 40 × 1 mm was produced from the virgin PP using an injection molding machine (GL-150, Sodick, Japan) with the conditions of polymer temperature 220 °C, injection speed 50.24 cm^3^/s and mold temperature 34 °C. The molded plate was then divided equally into two parts, as shown in Figure 1. The part near to the gate side is expressed as NG (near the gate), and the opposite side of the gate is expressed as FG (far from the gate) in the Figure. In the present study, we used the NG parts of the plates for conducting the melt spinning experiment, considering that the effect of flow history is reported to be higher for the NG part [19]. All the NG parts were crushed by a crusher machine to prepare small granules for melt spinning. From the following, we will indicate the polymer from the NG parts as ‘recycled PP’.

### 2.2. High-Speed Melt Spinning

**Figure 2:**
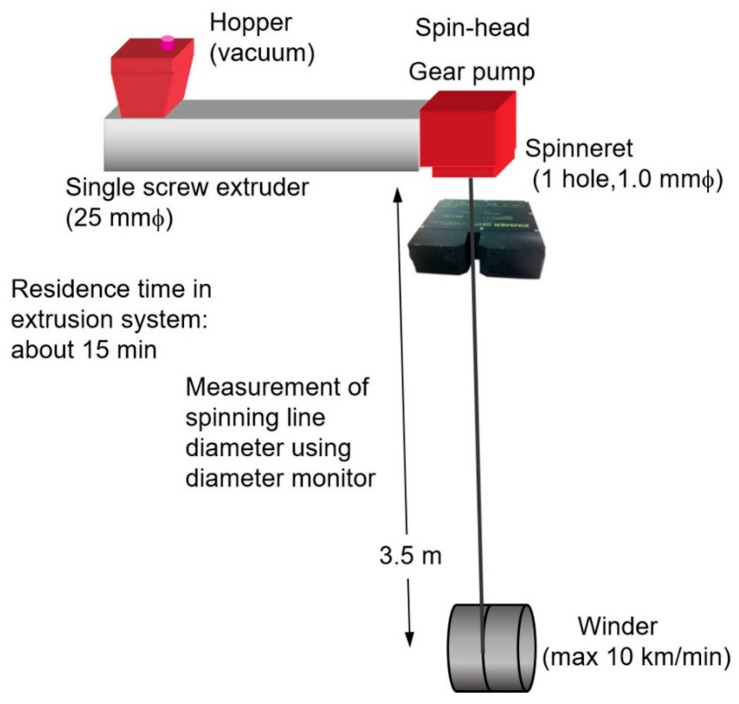
Schematic diagram of melt spinning apparatus.

The schematic diagram of the melt spinning apparatus consisting of an extrusion system and a winder is shown in Figure 2. PP was melted and extruded through a spinneret with a single hole of 1 mm diameter at 230 °C. The extruded fibers were taken up by a high-speed winder placed at 350 cm below the spinneret. No quenching air flow was applied. The throughput rate was maintained at 5.0 g/min, and take-up velocity was varied from 0.5 to 5.0 km/min. Fiber samples could not be obtained for over 5.0 km/min because of the breakage of the spin-line.

### 2.3. On-Line Measurement

To observe the thinning behavior of PP in the high-speed spin-line, on-line measurement of the diameter of spin-line was carried out using a noncontact, back-illumination type diameter monitor (Model 460-A/10, Zimmer OHG, Rheinau, Germany). The measurements were conducted from 10 to 300 cm down from the spinneret with an interval of 10 cm. The diameter versus frequency diagrams were prepared from the data acquired at each position to estimate the average diameter as well as the diameter fluctuation of the spin-line.

### 2.4. Analysis of As-Spun Fibers

#### 2.4.1. Tensile Test

The stress-strain curves for the PP fibers were measured by a tensile testing machine (SHIMADZU AG-I, Kyoto, Japan). The gauge length was 20 mm and tensile speed was 20 mm/min. Five samples were measured for fibers of each preparation condition.

#### 2.4.2. Birefringence Measurement

Refractive indices of fibers in the parallel and perpendicular directions with respect to the fiber axis were measured using an interference microscope (Carl-Zeiss Jena, Germany) equipped with a polarizing filter. Immersion liquids of various refractive indices were used to obtain proper interference fringes under the interference microscope. Birefringence *Δn* was calculated from the two refractive indices using the following Equation (1):

*Δn* = *n*_//_ − *n*_⊥_ (1)

where, *n*_//_ and *n*_⊥_ are the refractive indices of the fibers in the directions parallel and perpendicular to the fiber axis, respectively.

#### 2.4.3. Wide Angle X-ray Diffraction (WAXD) Analysis

Two-dimensional (2-D) WAXD patterns were obtained to analyze the crystalline structure of as-spun PP fibers. The WAXD intensity distribution measurement for the fiber bundles were performed by using a Ni-filtered CuKα radiation source (wavelength = 0.15418 nm) generated at 60 kV and 45 mA, and a mercury charged-coupled device (CCD) X-ray detector (Rigaku Co., Ltd., Tokyo, Japan) at a camera length of 35 mm.

## 3. Results and Discussion

### 3.1. Melt Spinning Behavior of Virgin and Recycled PP

Generally, in the melt spinning process at high take-up velocities, after gradual thinning of the spin-line, an abrupt and sharp decrease in diameter occurs at a certain point. This behavior is called ‘neck-like deformation’ [20,21,22,23,24,25,26,27]. Neck-like deformation is known to play an important role in the stabilization of the melt spinning process and the development of the fiber structure [25,26]. After completion of the neck-like deformation, the additional decrease in diameter is minimal. The position where the spin-line diameter reaches the final diameter is called the solidification point. Solidification of the spin-line of PP corresponds to the crystallization, and the shift of solidification point to upstream indicates the promotion of flow-induced or stress-induced crystallization.

**Figure 3:**
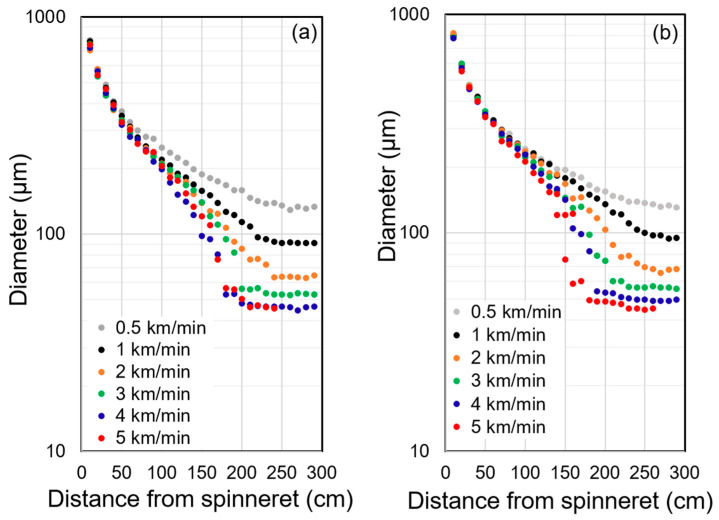
Diameter profiles of the melt spinning line for various take-up velocities; (**a**) virgin PP, (**b**) recycled PP.

Figure 3a,b show the diameter profiles for the spin-line from the virgin, and recycled PP, respectively. Diameter data obtained at each position were simply averaged and plotted in these Figures. For both kinds of spin-line, the final diameter decreased with the increased take-up velocity because the throughput rate was kept constant. In these figures, a smooth and gradual thinning was observed in the entire region of the spin-line for the take-up velocity of 0.5 km/min, and no ‘neck-like deformation’ was observed for both virgin and recycled PP. With the increase in take-up velocity, thinning of the spin-line was promoted mainly in the downstream, whereas the thinning behavior in the upstream, i.e., the region with the distance from the spinneret of about 0 to 50 cm, was not influenced by the take-up velocity. When the take-up velocity exceeded 2.0 km/min, the solidification point gradually shifted upstream (closer to the spinneret) of the spin-line with the increase in take-up velocity, and steep thinning was observed immediately before the solidification point. This behavior can be regarded as the ‘neck-like deformation’. The shape of the neck-like deformation became steeper at higher take-up velocities.

Comparing the thinning behaviors of virgin and recycled PP, a clear difference between the two polymers could not be found, partly because of a slight scatter of the data. At the take-up velocity of 1.0 km/min, however, the solidification point for the virgin PP appeared to be slightly closer to the spinneret than the recycled PP.

**Figure 4:**
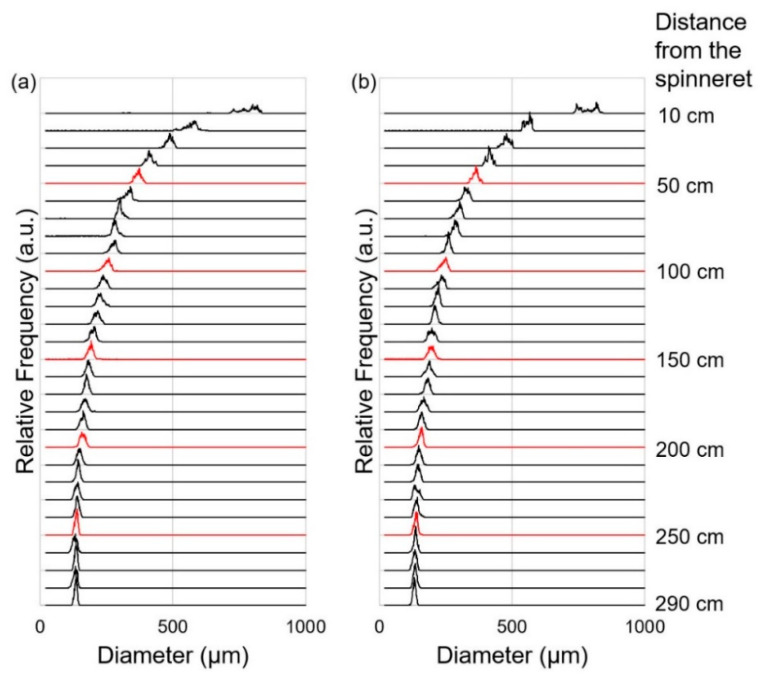
Diameter frequency diagrams measured for various positions of melt spinning line for the take-up velocity of 0.5 km/min; (**a**) virgin PP, and (**b**) recycled PP.

**Figure 5:**
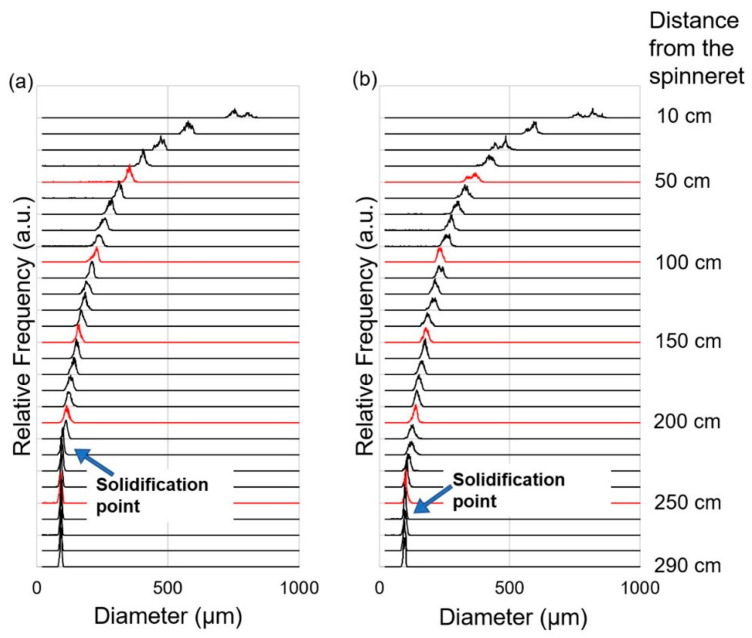
Diameter frequency diagrams measured for various positions of the melt spinning line for the take-up velocity of 1.0 km/min; (**a**) virgin PP, and (**b**) recycled PP.

In order to investigate the thinning behavior more in detail, we have prepared frequency distribution diagrams of diameter from the data acquired at each position of the spin-line. The results for the take-up velocities of 0.5 and 1.0 km/min are shown in Figure 4 and Figure 5, respectively. In general, the peak value and the width of the frequency distribution became lower and narrower with the increased distance from the spinneret. For the take-up velocity of 0.5 km/min, the difference between virgin and recycled PP was not clear. On the other hand, for the take-up velocity of 1.0 km/min, abrupt narrowing of the diameter distribution was observed at the positions of 220 cm for virgin PP and 240–260 cm for recycled PP. Below these positions, there was no further reduction of the peak value of diameter. It can be considered that the abrupt narrowing corresponds to the occurrence of neck-like deformation and starting of flow-induced crystallization [25]. From this analysis, it was verified that the flow-induced crystallization in the melt spinning process was suppressed for the recycled PP, which experienced a certain level of flow history in the injection molding process. It should be noted that only the NG part of injection molded product was used in this research, and the history effects were only verified for a part of the recycled polymer, which is considered to experience the highest degree of flow history.

### 3.2. Structure and Properties of As-Spun Fibers

**Figure 6:**
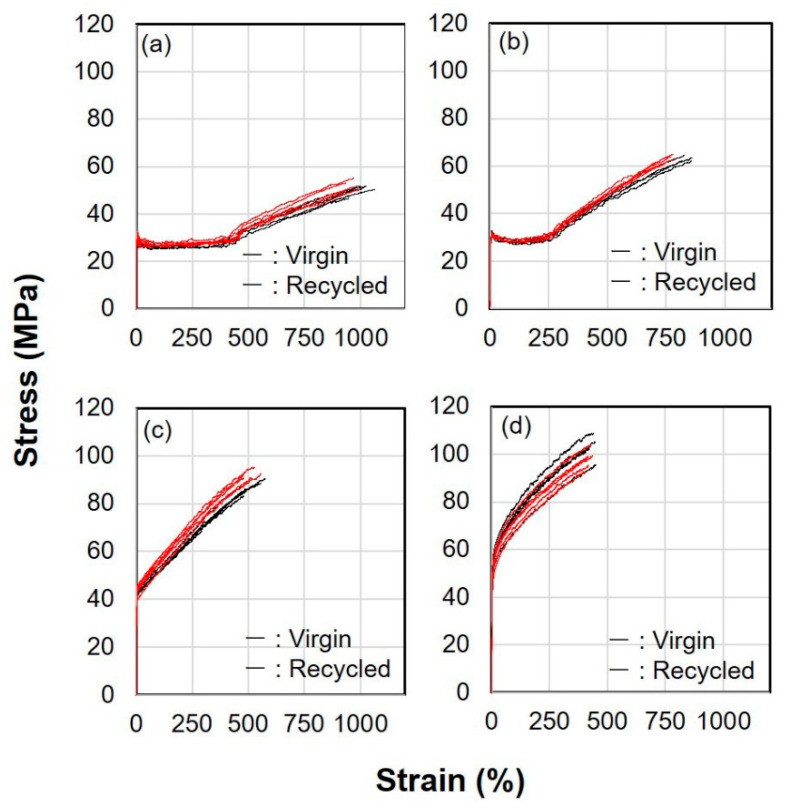
Stress–strain curves for the virgin and recycled PP fibers melt spun at different take-up velocities; (**a**) 0.5 km/min, (**b**) 1.0 km/min, (**c**) 3.0 km/min and (**d**) 5.0 km/min.

The structure and properties of high-speed spun fibers were investigated to verify the effect of flow history. Stress-strain (S-S) curves for the fibers from virgin and recycled PP of selected take-up velocities are shown in Figure 6. It was confirmed that the tensile strength increased and the elongation at break decreased as the take-up velocity increased. Region of constant stress found for the fibers of 0.5 and 1.0 km/min indicated the occurrence of necking deformation during the tensile testing. The natural draw ratio (necking draw ratio) decreased with the increase in take-up velocity. The latter half of the S-S curves showed that tensile strength increased steeply with the increased take-up velocity. Nevertheless, the difference between the fibers from virgin and recycled PP was not clear even for the take-up velocity of 1.0 km/min. In addition, Young’s modulus of the fibers, which cannot be estimated from Figure 6 because of the scale of abscissa, increased from around 1.1 to 1.8 GPa with the increases in take-up velocity from 0.5 to 5 km/min, however, the difference between the fibers of virgin and recycled PP could not be detected.

**Figure 7:**
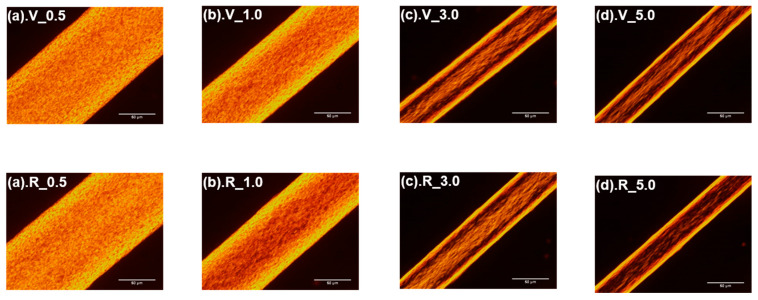
Optical micrographs observed under a polarizing microscope for the fibers from virgin (**upper row**) and recycled (**lower row**) PP, melt spun at different take-up velocities. The take-up velocities were (**a**) 0.5 km/min, (**b**) 1.0 km/min, (**c**) 3.0 km/min and (**d**) 5.0 km/min.

Figure 7 shows the polarizing microscope images of the fibers from virgin and recycled PP. For both kinds of fibers, although the fiber diameter became smaller as the take-up velocity increased, the primary interference fringes became clearer and separated, indicating that birefringence increased. However, no clear difference could be observed between the fibers from virgin and recycled PP.

**Figure 8:**
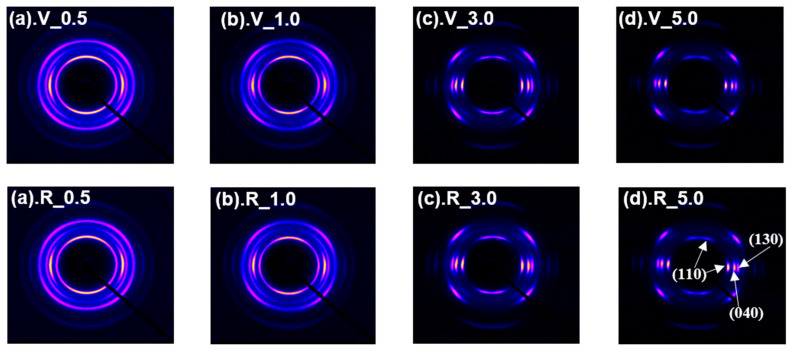
WAXD patterns for the fibers from virgin (**upper row**) and recycled (**lower row**) PP, melt spun at different take-up velocities. The take-up velocities were (**a**) 0.5 km/min, (**b**) 1.0 km/min, (**c**) 3.0 km/min and (**d**) 5.0 km/min.

WAXD analyses were performed to investigate the differences in the crystalline structure of fibers from virgin and recycled PP. Figure 8 shows the 2D WAXD patterns of the two kinds of fibers obtained at varied take-up velocities. For both kinds of fibers, the spread of crystalline reflections along an azimuthal angle gradually became narrower with the increased take-up velocity, which indicated that the degree of crystal orientation in the fibers increased with the increased take-up velocity.

It was reported that the pseudohexagonal form and/or α-form crystals develop in the melt spinning of isotactic PP depending on the processing conditions [28]. As can be seen from Figure 8, however, the formation of only α-form crystal was detected. This is mainly due to a relatively high content of ethylene components in the PP used in this research.

It is also well known that the crystals with two different types of orientation modes with respect to the fiber axis, i.e., *c*-axis orientation and a*-axis orientation, are formed in the PP fibers prepared through the melt spinning process. The latter is often called the epitaxially grown secondary (daughter) lamellae, whereas it was confirmed through the on-line WAXD measurement of the spin-line of PP that both types of orientation mode develop almost simultaneously in the melt spinning process [29].

The existence of the a*-axis oriented crystals can be detected through the appearance of (110) reflection (the innermost crystalline reflection in the 2-D WAXD pattern) in the meridional direction in addition to the (110) reflection on the equator, which corresponds to the *c*-axis oriented crystals. Both the fibers from virgin and recycled PP were found to have the crystals of both orientation modes, and the reflection from the a*-axis oriented crystals became less distinct with the increase in take-up velocity.

**Figure 9:**
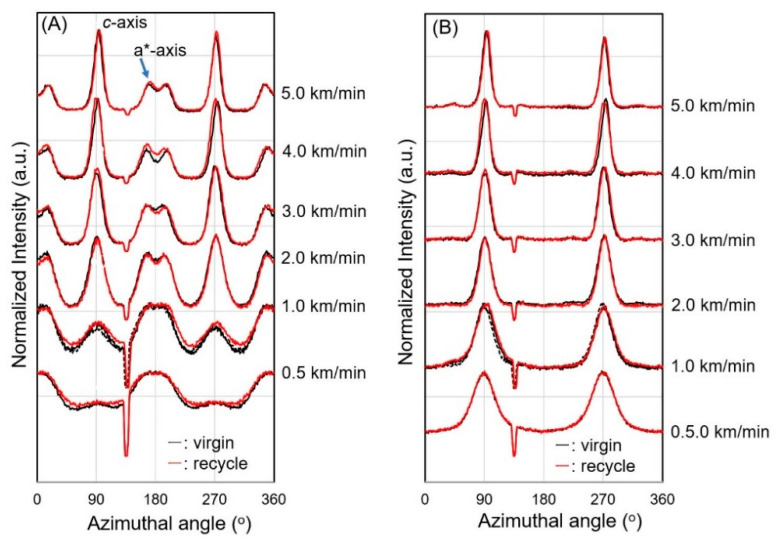
Azimuthal intensity distributions for (**A**) (110) and (**B**) (040) reflections observed through WAXD measurements for virgin PP and recycled PP fibers, melt-spun at different take-up velocities.

Now at this stage, though the WAXD patterns for each identical take-up velocity for the fibers from virgin and recycled PP resembled each other, we found some differences in the WAXD azimuthal profiles of fibers from virgin and recycled PP for the (110) and (040) reflection intensities as shown in Figure 9. For the (110) reflection, both equatorial and meridional reflections at the azimuthal angles of 0 (180) degrees and 90 (270) degrees were detected. The intensity of equatorial reflection became higher, and that of meridional reflection became lower with the increase in take-up velocity. The degree of crystalline orientation, which can be judged from the narrowing of the width of reflection, also increased with the take-up velocity. On the other hand, only equatorial reflection was observed for (040) because reflections from both *c*-axis oriented and a*-axis oriented crystals appeared on the equator. Nevertheless, the width of the peak decreased with the increased take-up velocity, indicating the increase in the degree of crystalline orientation.

Although the difference between the profiles for the fibers of virgin and recycled PP was not clear as expected, a noticeable difference was observed for the take-up velocity of 1.0 km/min, where the peak intensity for the *c*-axis oriented crystals was stronger and the widths for the *c*-axis and a*-axis oriented crystals were narrower for the fibers from virgin PP than those from recycled PP. A similar but less distinct difference was found even for the 0.5 km/min fibers. These results indicated that the relative amount of *c*-axis oriented crystal was lower, and the crystalline orientation was lower in the fibers from recycled PP in comparison with those from virgin PP. The tendency found in this analysis agrees with the above results of Figure 4 and Figure 5, i.e., the thinning completion point of the fibers from virgin PP is on the upstream side of the spin-line. The detailed mechanism for such change in crystalline orientation mode and degree of crystalline orientation is not clear. The lowering of the solidification point corresponds to the lowering of the crystallization temperature, which may suppress the crystallization-induced development of crystalline orientation [30]. From the results of higher take-up velocities, it was not possible to find any observable difference between the azimuthal profiles for the fibers from virgin and recycled PP.

**Figure 10:**
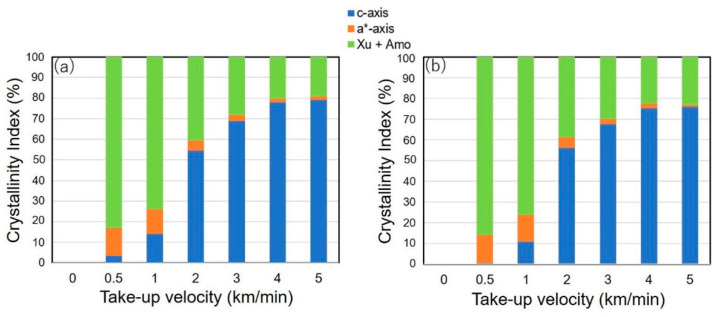
Changes of crystallinity index for a*-axis oriented crystals (■), c-axis oriented crystals (■) and the combined amount of unoriented crystals and amorphous phase (■) with increased take-up velocity for the fibers from (**a**) virgin and (**b**) recycled PP, melt spun at different take-up velocities.

The amount of a*-axis crystal in a melt-spun fiber may have a considerable effect on the fiber properties. We were interested in how the amounts of components (*c*-axis oriented crystals and a*-axis oriented crystals) in the fibers changed at varied take-up velocities. Azimuthal intensity distribution curve for (110) was separated into three components, i.e., a peak on the equator, a peak on the meridian and an isotropic background, and relative amounts of *c*-axis oriented crystal, a*-axis oriented crystal and (unoriented crystal + amorphous) components were obtained assuming the axis-symmetric structure in the fibers. Figure 10 shows the relative amount of the three components (crystallinity index) in the fibers of virgin PP and recycled PP prepared at varied take-up velocities. It was found that though a significant amount of a*-axis oriented crystals was present at lower take-up velocities of 0.5 and 1.0 km/min, the amount gradually decreased with increased take-up velocity and finally became almost negligible at the take-up velocity of 5.0 km/min. It was also found that for the take-up velocities of 0.5 and 1.0 km/min, the amount of *c*-axis oriented crystals was obviously higher for the fibers from virgin PP compared to the fibers from recycled PP. This result also coincides with the results shown in Figure 4 and Figure 5, in that a higher tendency for the occurrence of flow-induced crystallization in virgin PP was presented.

It has been reported that the molding process induces distortion of chain conformation [31,32], warpage and shrinkage deformations [33], and changes in crystal and inner structure [16]. It was also discussed that the number of tie molecules in the recycled polymer decreases due to the history of the injection molding process [19]. Polymers have entanglements in the molten state. In semicrystalline polymers, the entanglement density may correlate with the number of tie molecules in the crystallized structure, which is supposed to influence their deformation behavior and mechanical properties.

From the above discussion, we speculated that the reduction of the density of chain entanglement caused by the shear flow applied during the injection molding process was significant enough to affect the melt spinning behavior of recycled PP, in that suppression of crystallization compared to the virgin PP occurred in the mild elongational-flow in the low take-up velocity region. This presumably yielded the difference in the higher-order structure of crystalline orientation in the fibers of the virgin and recycled PP.

At higher take-up velocities, however, the difference in the spinning behavior between the virgin and recycled PP could not be detected mainly due to the fluctuation of the position of neck-like deformation. The stronger effect of flow with a much higher elongational strain rate in the melt spinning process may also prevent the detection of the difference in flow-induced crystallization behavior. Accordingly, the effect of processing history on the higher-order structure as well as on the mechanical properties of the resultant fibers did not become noticeable.

## 4. Conclusions

Verification of the effect of processing history on the properties of recycled polymer products was attempted by investigating the high-speed melt spinning behavior of virgin and recycled PP, where the ‘model’ recycled PP was prepared through the injection molding of virgin PP. On-line measurement of the thinning behavior of the spin-line revealed the downstream shift of solidification point for the recycled PP at the take-up velocity of 1.0 km/min, indicating the suppression of flow-induced crystallization. Deterioration of flow-induced crystallization was also suggested through the detailed investigation of the variation of the relative amount of *c*-axis– and a*-axis–oriented crystals in the fibers prepared at varied take-up velocities. It was speculated that the history of the injection molding process induced the lowering of the number of molecular entanglements, which affected the melt spinning behavior and crystallization process. The difference between the virgin and recycled PP became undistinguishable with increased take-up velocity. This result suggested the existence of an optimum elongational strain rate for the detection of the different states of molecular entanglement.

## Data Availability

Not applicable.
